# The presence of headache at onset in SARS-CoV-2 infection is associated with long-term post-COVID headache and fatigue: A case-control study

**DOI:** 10.1177/03331024211020404

**Published:** 2021-06-16

**Authors:** César Fernández-de-las-Peñas, Víctor Gómez-Mayordomo, María L Cuadrado, Domingo Palacios-Ceña, Lidiane L Florencio, Angel L Guerrero, David García-Azorín, Valentín Hernández-Barrera, Lars Arendt-Nielsen

**Affiliations:** 1 Department of Physical Therapy, Occupational Therapy, Physical Medicine and Rehabilitation, Universidad Rey Juan Carlos, Madrid. Spain; 2 Department of Neurology, Hospital Clínico San Carlos, Madrid, Spain; 3 Department of Medicine, School of Medicine, Universidad Complutense de Madrid, Madrid, Spain; 4 Headache Unit, Department of Neurology, Hospital Clínico Universitario de Valladolid, Valladolid, Spain; 5 Neuroscience Research Unit, Institute for Biomedical Research of Salamanca, Salamanca, Spain; 6 Department of Medicine, Universidad de Valladolid, Valladolid, Spain; 7 Department of Public Health, Universidad Rey Juan Carlos, Madrid. Spain; 8 CNAP, Center for Sensory-Motor Interaction, Department of Health Science and Technology, Faculty of Medicine, Aalborg University, Aalborg, Denmark

**Keywords:** COVID-19, headache, fatigue, post-COVID, anxiety, depression, sleep

## Abstract

**Objective:**

To investigate the association of headache during the acute phase of SARS-CoV-2 infection with long-term post-COVID headache and other post-COVID symptoms in hospitalised survivors.

**Methods:**

A case-control study including patients hospitalised during the first wave of the pandemic in Spain was conducted. Patients reporting headache as a symptom during the acute phase and age- and sex-matched patients without headache during the acute phase participated. Hospitalisation and clinical data were collected from medical records. Patients were scheduled for a telephone interview 7 months after hospital discharge. Participants were asked about a list of post-COVID symptoms and were also invited to report any additional symptom they might have. Anxiety/depressive symptoms and sleep quality were assessed with the Hospital Anxiety and Depression Scale and the Pittsburgh Sleep Quality Index.

**Results:**

Overall, 205 patients reporting headache and 410 patients without headache at hospitalisation were assessed 7.3 months (Standard Deviation 0.6) after hospital discharge. Patients with headache at onset presented a higher number of post-COVID symptoms (Incident Rate Ratio: 1.16, 95% CI: 1.03–1.30). Headache at onset was associated with a previous history of migraine (Odd Ratio: 2.90, 95% Confidence Interval: 1.41–5.98) and with the development of persistent tension-type like headache as a new post-COVID symptom (Odd Ratio: 2.65, 95% CI: 1.66–4.24). Fatigue as a long-term symptom was also more prevalent in patients with headache at onset (Odd Ratio: 1.55, 95% CI: 1.07–2.24). No between-group differences in the prevalence of anxiety/depressive symptoms or sleep quality were seen.

**Conclusion:**

Headache in the acute phase of SARS-CoV-2 infection was associated with higher prevalence of headache and fatigue as long-term post-COVID symptoms. Monitoring headache during the acute phase could help to identify patients at risk of developing long-term post-COVID symptoms, including post-COVID headache.

## Introduction

Clinical manifestations associated with severe acute respiratory syndrome coronavirus 2 (SARS-CoV-2) infection are heterogeneous and can include respiratory (dyspnoea, chest pain, cough), gastrointestinal (diarrhoea, vomiting) and musculoskeletal (myalgias, joint pain) symptoms. Additionally, neurological manifestations such as headache, dizziness, anosmia, or ageusia are likewise symptoms of coronavirus disease 2019 (COVID-19) (1,2). A meta-analysis reported a headache prevalence of 10.1% (95% CI 8.76–11.49) in COVID-19 patients (3). Headache has been also found to be an early-onset symptom of COVID-19 disease and can also emerge as an isolated symptom (4).

Although headache associated with SARS-CoV-2 infection should be diagnosed as “headache attributed to systemic viral infection” (5), its features resemble migraine or tension-type headache, without a predominant pattern (6,7). Some studies have reported that the presence of headache as an onset symptom of SARS-CoV-2 infection is associated with a more benign clinical course of the disease (8,9). However, no data on the association of headache at onset with persistent headache or other post-COVID symptoms in the long term is available. Nowadays, the world is in front of a second pandemic associated with COVID-19: post-COVID sequelae. Different terms are used to describe the presence of post-COVID symptoms, with “long COVID” being the most commonly used. “Long COVID” refers to patients who have recovered from COVID-19 but who continue to have symptoms for much longer than would be expected (10). There is evidence suggesting that 75% of COVID-19 survivors exhibit post-COVID sequelae (11–13). Several studies have investigated the presence of a myriad of post-COVID symptoms and have observed prevalence rates ranging from 2% to 15% for headache (11–14). However, no specific data on post-COVID headache and its features were provided. Additionally, no studies have examined the possible relationship between acute phase headache and the presence of post-COVID symptoms in the long-term.

We investigated the potential association between the occurrence of headache during the acute COVID-19 phase in previously hospitalised patients and the presence of post-COVID headache and other long-term post-COVID symptoms after 6 months. We hypothesised that the occurrence of headache at the onset of SARS-CoV-2 infection would be associated with a higher prevalence of persistent headache and other post-COVID symptoms in the long term.

## Methods

### Participants

A case-control study including individuals hospitalised during the first wave of the pandemic in Madrid who had recovered from acute SARS-CoV-2 infection was conducted. All participants were positively diagnosed of SARS-CoV-2 infection with real-time reverse transcription-polymerase chain reaction (PCR) assay of nasopharyngeal and/or oral swab samples and the presence of consistent clinical and radiological findings at hospitalisation. Participants should have been discharged without rehospitalisation at the time of the study. Subjects with medical diagnosis of dementia, delirium or psychiatric conditions (or otherwise unable to conduct the interview) were excluded. The study was approved by the Ethics Committees of Hospital Clínico San Carlos and Universidad Rey Juan Carlos (HCSC20/495E, URJC0907202015920). All participants were informed of the study and provided verbal informed consent before collecting any data.

Demographic and clinical admission data were collected from medical records, including age, gender, height, weight, pre-existing medical comorbidities, intensive care unit [ICU] admission, days at hospital, and symptoms at hospitalisation. Prior history of headache was assessed, and it was considered if the diagnosis was previously established by a neurologist.

Of all the patients hospitalised due to SARS-CoV-2 infection between February 20 and May 31, 2020 in Hospital Clínico San Carlos (Madrid, Spain), those who reported headaches as a symptom during hospital admission were selected as cases. In addition, two sex- and age-matched controls not reporting headache at hospital admission were recruited for each case. If more than two controls per case were available in the hospital records, selection was randomised using a random number generator (Random.org).

### Procedure

Participants who agreed to participate were scheduled for a telephone semi-structured interview by trained researchers following procedures used in population-based survey studies. Interviews were conducted by specialised healthcare professionals blinded to the patient condition (case, control). To maintain blinding in the assessment, the first part of the interview focused on post-COVID symptoms, and questions related to symptoms in the acute phase were asked afterwards. First, participants were asked to report the presence of symptoms appearing after hospitalisation, and whether the symptom persisted at the time of the interview. It was emphasised that these symptoms should have appeared after infection (post-COVID related symptom). They were systematically asked for a predefined list of post-COVID symptoms (i.e., dyspnoea, fatigue, headache, anosmia, ageusia, chest pain, palpitations, diarrhoea, cough, brain fog, and loss of concentration), but were completely free to report any symptom that they considered. In patients with post-COVID headache, headache characteristics, e.g., location, quality, intensity, and accompanying symptoms, were systematically recorded. Headache phenotypes were eventually classified according to the ICHD-3 criteria (5) by two neurologists. The interview was completed with questions about the symptoms that the patients had experienced in the acute phase of the infection, in particular headache, which were subsequently cross-checked with data from the medical records.

Finally, the Hospital Anxiety and Depression Scale (HADS) and the Pittsburgh Sleep Quality Index (PSQI) were used to assess anxiety/depressive symptoms and sleep quality, as both questionnaires can be properly administered by telephone interview (15). Briefly, the HADS includes one scale assessing anxiety (HADS-A, 7-items) and one scale assessing depressive (HADS-D, 7-items) symptoms. Each item is scored on a Likert scale (0-3) providing a maximum score of 21 points for each subscale (16). Although a cut-off score of ≥8 points on each subscale has shown good sensitivity and specificity (17), we considered the cut-off scores recommended for Spanish population, HADS-A≥12 points and HADS-D≥10 points, indicative of clinical anxiety and depressive symptoms, respectively (18). This questionnaire has shown good validity and reliability in the general population (19), and has been previously used for determining the presence of anxiety and depressive symptoms in COVID-19 patients during hospitalization (20).

The PSQI evaluates the quality of sleep over the previous month by including 19 self-rated questions assessing the usual bedtime, usual wake time, number of hours slept, and number of minutes to fall asleep (21). Questions are answered on a 4-point Likert-type scale (0-3), and the sum of all answers is transformed into a global score ranging from 0 to 21 points, where higher scores indicate worse sleep quality. A total score ≥8 points is indicative of poor sleep quality (21). The PSQI has shown good internal consistency and test-retest reliability (22).

### Statistical Analysis

The statistical analysis was conducted with STATA 16.1 (StataCorp. 2019. Stata Statistical Software: Release 16. College Station, TX: StataCorp LP, USA). Data were presented as mean (standard deviation, SD) or percentages as appropriate. The McNemar test and paired Student t-tests were conducted to compare proportions and means between patients with (cases) and without (controls) headache as symptom during the acute phase. Multivariable conditional logistic regression models were constructed to identify those variables related to hospitalisation (number of symptoms at hospitalisation, days at hospital, pre-existing medical comorbidities, ICU admission) and post-COVID symptoms associated with the presence of headache as symptom during the acute phase. Adjusted Odd Ratio (OR) or Incident Rate Ratios (IRR) with 95% confidence intervals (95% CI) were calculated. A priori the level of significance was set at 0.05.

## Results

Among 1,100 patients hospitalised due to SARS-CoV-2 infection from February 20 to May 31, 2020, a total of 205 (18.6%) reported headache as a symptom during the acute phase. Headache occurred within 48-72 hours of the first COVID-19 symptom and was described as very intense bilateral pain by most patients. Of these 205 patients, 60% (n = 123) were female and 40% (n = 82) were male, and the mean age was 55.5 years (SD: 14; range: 36-86 years). In addition, 410 age- and sex-matched hospitalised patients not reporting headache during the acute phase of the disease were taken as controls.

The most common symptoms at hospitalisation were fever, myalgias, dyspnoea, and cough. Dyspnoea and cough were less frequent in patients presenting with headache (21.4% and 17.5%, vs. 37.8% and 32%, respectively; P = 0.01). No other significant differences in onset symptoms were found (Table 1). Two hundred and ninety-four (48%) patients had no medical comorbidities, 288 (47%) had one or two comorbidities, and the remaining 31 (5%) reported ≥3 comorbidities. Patients with previous diagnosis of migraine were more prone to suffer from headache during the acute phase (14.1% vs. 7.8% in those without history of migraine; OR: 2.10, 95% CI: 1.19-3.73; P = 0.01). Demographic and hospitalisation data are summarised in Table 1.

**Table 1.** Demographic and hospitalisation data of COVID-19 patients with headache (cases) and without headache (controls) at onset.

	Cases (n = 205)	Controls (n = 410)
Age, mean (SD), years	55.5 (14.0)	55.4 (14.0)
Gender, male/female (%)	82 (40%) / 123 (60%)	164 (40%) / 246 (60%)
Weight, mean (SD), kg.	73.6 (12.5)	73.5 (15)
Height, mean (SD), cm.	164.0 (10)	165 (10.5)
Body Mass Index, mean (SD), kg/cm^2^	27.5 (4.5)	27.0 (5.0)
Smoking status, n (%)		
Active	18 (8.7%)	33 (8%)
None or Former	187 (91.3%)	376 (92%)
Symptoms at hospital admission, n (%)		
Headache	205 (100%)	0 (0%)
Fever	135 (65.8%)	304 (74.1%)
Myalgia	79 (38.5%)	160 (39%)
Dyspnoea*	44 (21.4%)	155 (37.8%)
Cough*	36 (17.5%)	131 (32%)
Diarrhoea	25 (12.2%)	54 (13.1%)
Anosmia	11 (5.4%)	47 (11.5%)
Ageusia	11 (5.4%)	34 (8.3%)
Throat Pain	11 (5.4%)	17 (4.2%)
Number of medical comorbidities, n (%)		
None	105 (51.2%)	191 (46.6%)
1 or 2	95 (46.3%)	193 (47.1%)
3 or more	5 (2.5%)	26 (6.3%)
Medical co-morbidities		
Hypertension	42 (20.5%)	89 (21.7%)
Diabetes	20 (9.7%)	37 (9%)
Cardiovascular Disease	16 (7.8%)	43 (10.5%)
Rheumatological Disease	4 (2%)	7 (1.7%)
Asthma	12 (5.8%)	31 (7.5%)
Obesity	10 (4.9%)	19 (4.6%)
Chronic Obstructive Pulmonary Disease	3 (1.4%)	16 (4%)
Stroke	5 (2.4%)	12 (3%)
Migraine*	29 (14.1%)	32 (7.8%)
Other (Cancer, Kidney Disease)	28 (13.6%)	55 (13.4%)
Stay at the hospital, mean (SD), days	12 (10.0)	13 (11.5)
Intensive Care Unit (ICU) admission		
Yes/No, n (%)	10 (4.8%)/195 (95.2%)	24 (5.8%)/386 (94.2%)
Stay at ICU, mean (SD), days	22 (13)	13 (14)

n: number; SD: Standard Deviation

* Statistically significant differences between cases and controls (P = 0.01)

Participants were assessed a mean of 7.3 months (SD: 0.6) after hospital discharge. At the time of the evaluation, from the total sample 103 (16.7%) were completely free of any post-COVID symptom, 251 (40.8%) had one or two symptoms, and the remaining 261 (42.5%) had ≥3 post-COVID symptoms. A greater proportion (*X^2^*: 14.141, P = 0.045) of patients reporting headache during the acute phase experienced ≥3 persistent post-COVID symptoms when compared to those without headache at onset. In fact, the number of post-COVID symptoms in the headache group (mean: 2.4, SD: 1.4) was significantly greater (IRR 1.16, 95% CI: 1.03–1.30; P = 0.012) than the number of post-COVID symptoms in the non-headache group (mean: 2.0, SD: 1.5).

**Figure 1.**
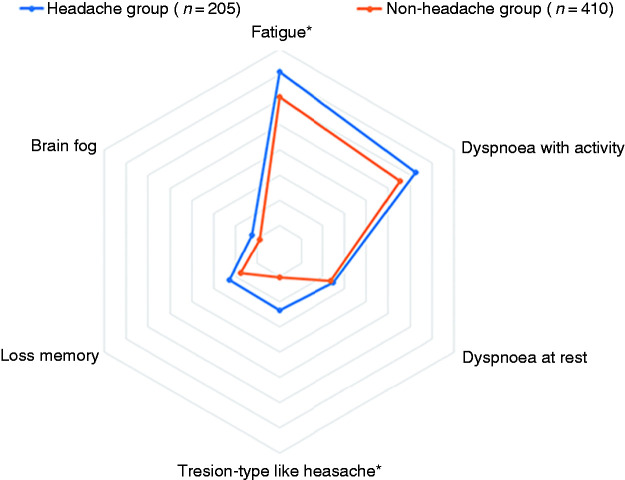
Distribution of the most prevalent post-COVID symptoms (fatigue, dyspnoea with activity, dyspnoea at rest, memory loss, brain fog, and tension-type like headache) in COVID-19 patients with and without experiencing headache as a symptom of COVID-19 at the acute phase of the disease.

Figure 1 graphs the distribution of the six most prevalent post-COVID symptoms 7 months after hospital discharge (i.e., fatigue, dyspnoea at exercise, dyspnoea at rest, memory loss, brain fog, and tension-type like headache) in patients with and without headache during the acute phase of the disease.

Post-COVID headache usually adopted features similar to those of tension-type like headache. When compared to those not presenting headache at the acute stage, a significant greater proportion of patients exhibiting headache as an acute COVID-19 symptom described the presence of tension-type like headache as a new post-COVID symptom (23.4% versus 10.5%; OR: 2.61 95% CI: 1.64–4.14; P < 0.001). This tension-type like headache was described as a bilateral pain, predominantly localised over the temporoparietal or frontal regions, usually of pressing quality and with moderate to severe intensity. Just a minority of patients had migraine-like headache as post-COVID symptom, with no between-group differences (OR: 0.99, 95% CI: 9.33-2.96; P = 0.95). This migraine-like headache was experienced as a unilateral pain, localised in the ocular and/or frontal region, of throbbing quality (sometimes pressing) and severe intensity, commonly accompanied by photophobia and/or phonophobia.

Among patients with previous history of migraine, 58.6% of those experiencing headache during the acute COVID-19 phase reported a worsening of their migraine after the infection. In contrast, 34.3% of the migraineurs not experiencing headache during the acute phase had worsening of their migraine after the infection (P < 0.001). Thirty-seven patients (60.6%) experienced an increase in the frequency of migraine, 19.7% (n = 12) an increase in the intensity, and the remaining 19.7% (n = 12) in both frequency and intensity (Table 2). Only one patient with a prior diagnosis of migraine developed tension-type like headache as a new symptom after COVID-19. In fact, a previous history of migraine was not associated with the development of post-COVID tension-type like headache (OR 1.08, 95% CI: 0.41–2.87; P = 0.869).

**Table 2.** Prevalence of post-COVID symptoms in patients with (cases) and without (controls) headache in the acute phase of infection.

	Cases (n=205)	Controls (n=410)
Number of post-COVID symptoms, n (%)*		
None	21 (10.2%)	82 (20%)
1 or 2	87 (42.4%)	164 (40%)
3 or more	97 (47.4%)	164 (40%)
Post-COVID symptoms, n (%)		
Fatigue	145 (70.7%)	249 (60.7%)
Dyspnoea with activity	128 (62.4%)	228 (55.6%)
Dyspnoea rest	51 (24.9%)	96 (23.3%)
Tension-Type Like Headache*	48 (23.4%)	43 (10.5%)
Memory loss	47 (23%)	72 (17.5%)
Cognitive Blunting - Brain fog	26 (12.7%)	36 (8.8%)
Gastrointestinal Disorders - Diarrhoea	19 (9.2%)	47 (11.5%)
Tachycardia-Palpitations	19 (9.2%)	28 (6.8%)
Ocular/Vision Disorders	23 (11.2%)	29 (7.1%)
Ageusia/Hypogeusia	8 (3.9%)	16 (3.9%)
Anosmia/Hyposmia	8 (3.9%)	16 (3.9%)
Cough	7 (3.4%)	6 (1.4%)
Dizziness	6 (3%)	8 (1.9%)
Migraine Like Headache	5 (2.4%)	10 (2.4%)
Worsening of Previous Migraine		
Yes/No, n (%)	17 (58.6%) / 12 (41.4%)	11 (34.3%) / 21 (65.7%)
Frequency Increase	17 (58.6%)	20 (62.5%)
Intensity Increase	5 (17.2%)	7 (21.9%)
Frequency and Intensity Increase	7 (24.2%)	5 (15.6%)
HADS-D (0-21), mean (SD)	5.0 (4.7)	5.2 (5.0)
Depressive Symptoms (HADS-D ≥10 points), n (%)	47 (23%)	91 (22.2%)
HADS-A (0-21), mean (SD)	5.2 (5.0)	5.4 (5.3)
Anxiety Symptoms (HADS-A ≥12 points), n (%)	29 (14.2%)	69 (16.8%)
PSQI (0-21), mean (SD)	7.0 (4.0)	6.5 (4.0)
Poor Sleep Quality (PSQI ≥8 points), n (%)	79 (38.5%)	138 (33.6%)

HADS: Hospital Anxiety and Depression Scale (A: Anxiety; D: Depression);

PSQI: Pittsburgh Sleep Quality Index; SD: Standard Deviation

* Statistically significant differences between cases and controls (P < 0.05)

Of the remaining post-COVID symptoms, fatigue was the only one that showed significant between-group differences. Indeed, persistent fatigue was more common in patients who had headache in the acute phase as compared to those who did not have headache (OR: 1.55, 95% CI: 1.07–2.24; P = 0.02). No significant differences in the presence dyspnoea at rest, dyspnoea with activity, memory loss or cognitive blunting as post-COVID symptoms were seen depending on the presence or absence of headache in the early stage of the disease (Table 2). There were also no between-groups differences in the scores on HADS-A (P = 0.704), HADS-D (P = 0.608), and PSQI (P = 0.441). In fact, there was no association between the occurrence of headache as a symptom at onset and the detection of depression (OR: 1.04, 95% CI: 0.70–1.54; P = 0.84), anxiety (OR: 0.82, 95% CI: 0.51–1.30; P = 0.397) or poor sleep quality (OR: 1.24, 95% CI: 0.87–1.75; P = 0.233) after 7 months (Table 2).

The multivariate analysis revealed that, after adjusting for all variables, the presence of a history of pre-existing migraine (OR: 2.90, 95% CI: 1.41–5.98; P = 0.004) and the development of post-COVID tension-type like headache (OR: 2.65, 95% CI: 1.66-4.24; P < 0.001) were those that were independently associated with suffering from headache at onset.

## Discussion

This study found that 83% of previously hospitalised COVID-19 survivors exhibited at least one post-COVID symptom seven months after hospital discharge. The presence of headache as a symptom at hospitalisation was associated with persistent headache and fatigue as long-term post-COVID symptoms. No differences in anxiety/depressive levels or sleep quality between patients who did or did not have headache at hospitalisation were found.

Evidence supports that COVID-19 patients can exhibit a plethora of symptoms at infection, with fever, cough, fatigue, and dyspnoea being the most prevalent (23). In previous studies, the presence of headache as an onset symptom was significantly associated with anosmia/ageusia (24) or fever (25); however, we did not find such association. In agreement with our findings, Rubio-Rivas et al. identified 4 clusters of COVID-19 patients where those predominantly presenting with ageusia or anosmia and those predominantly with headache and myalgias represented different groups (26). The COVID‐19 Task Force of the International Federation of Otorhinolaryngological Societies found that headache, nasal obstruction, and anosmia were the most prevalent symptoms at onset (27), results totally different from those reported by Alimohamadi et al (23). These results would support that the heterogeneity commonly seen in the clinical presentation of COVID-19 is also found within the headache spectrum, since no clear presentation is predominant (6,7). In agreement with this hypothesis, Planchuelo-Gómez et al. described two different COVID-19 related phenotypes of headache, i.e., tension-type like and migraine-like headache (28). We found that the presence of headache as a symptom in the acute phase was more prevalent in patients with pre-existing migraine, which may have important clinical implications for clinical examination. Our results agree with previous studies supporting that previous history of headache is more common in people presenting headache as an onset symptom, but, obviously, not exclusive (9,25). The phenotype of headache as a symptom at COVID-19 onset is usually perceived as different from the pre-existing usual headache (29).

In our particular sample, we found that the prevalence of headache as a long-term post-COVID symptom was 15% in hospitalised patients. Our data are similar to those previously reported by Carfi et al. (11), higher than those reported by Arnold et al. (12), Huang et al. (13), and Logue et al. (14) reporting a prevalence of headache of 3-5%, and much lower than those observed by Gonzalez-Martinez et al., with a reported prevalence of 30% (9). Differences in the population sample, age, comorbidities, previous headache history, time from hospital discharge, or clinical course of the disease could explain the discrepancies. There is a need for multicentre studies including large populations that systematically examine headache as a post-COVID symptom.

Two previous studies have reported that the occurrence of headache as a symptom at onset of SARS-CoV-2 infection is associated with a more benign clinical course of the disease at the hospitalisation phase (8,9). Our results suggest that the presence of headache at hospital admission was associated with a greater prevalence of post-COVID headache and fatigue seven months after hospital discharge. Identification of individuals at risk of developing post-COVID headache could lead to better therapeutic strategies. The fact that headaches, particularly migraine, and COVID-19 share common underlying mechanisms (see below) supports that the presence of this symptom at the acute phase of the infection could promote the occurrence of post-COVID symptoms. In such a scenario, the presence of acute headache could correspond to a cascade of events that, in predisposed patients, would lead to the development of systemic (i.e., fatigue) but also specific (i.e., headache) post-COVID symptoms. This is an important topic since headache is not considered one of the most bothersome symptoms experienced by individuals at the acute COVID-19 phase, when compared with other symptoms such as dyspnoea or fever. Consequently, it is possible that headache is underreported by patients at hospital admission. Our results support the relevance of properly identifying, and maybe early managing, acute headache in COVID-19, given its association with persistent and disabling symptoms. Obviously, the development of post-COVID headache is not exclusive to those patients experiencing this symptom at onset, since a minority of patients not experiencing acute headache will also experience long-term post-COVID headache.

Interestingly, with the exception of one patient, none of the patients developing tension-type like headache as a new symptom after COVID had a prior diagnosis of migraine. These results are in line with those of Caronna et al., who found that 50% of COVID-19 patients presenting with headache as post-COVID symptom 6 weeks after hospital discharge had no previous history of headache (8). These results suggest that this headache is directly related to the infection itself. Indeed, post-COVID related headache in those patients without previous headache history could emerge as a new persistent headache of post-infectious aetiology. This pattern of emergence could meet criteria for new daily persistent headache (NDPH), which is daily and unremitting from its onset (30,31). Remarkably, other past viral outbreaks also provoked daily persistent headache (32). In our sample, we have not precisely characterised the temporal pattern of post-COVID headache, since we collected data cross-sectionally. We have also not collected data on the treatments used for this headache and the response to treatments. Further studies are now needed to characterise this potential new COVID-19 related headache and if specific management options are required.

Several hypotheses explaining the association between headache and SARS-CoV-2 infection can be proposed. There is evidence of a prolonged pro-inflammatory response (cytokine storm) in COVID-19 patients which can lead to a rapid hyperactivation of T cells, macrophages, and natural killer cells, and the overproduction of >150 inflammatory mediators (33). In such a situation, the COVID-related cytokine storm could promote several mechanisms associated with headaches: 1, an atypical response of the mast cells (34); 2, a dramatic increase of interleukin-6 (IL-6) levels (35); 3, over-expression of the angiotensin-converting enzyme 2 (ACE2) at central and peripheral nervous systems (36). Interestingly, the same mechanisms, i.e., hyper-responses of mast cells (37), neuroinflammation by IL-6 (38), and hyper-activation of ACE2 receptors (39), also play a relevant role in tension-type and migraine headaches. It is possible that these cytokine storm responses could lead to hyper-excitability of the trigemino-vascular system throughout different pathways promoting the development of headache at onset or as a post-COVID symptom. In this context, the headache in the acute phase may be followed by persistent headache or worsening of a pre-existing headache in predisposed individuals. Both clinical scenarios were observed in our study. Further, other factors, e.g., altered microstructural and functional integrity of the brain or emotional distress seen in COVID-19 survivors, could also promote the development of post-COVID headache. We did not observe differences in the presence of anxiety/depressive levels and poor sleep quality depending on the presence or absence of headache as a symptom at COVID-19 onset. However, we were unable to compare emotional factors between patients who developed post-COVID headache and those who did not due to sample size limitations. Future studies are needed to determine underlying mechanisms that explain the heterogeneity of headache in these patients in order to select appropriate treatments for this condition.

The results of this study should be considered taking into account some weaknesses. First, we only included hospitalised COVID-19 survivors; therefore, we cannot extrapolate our results to non-hospitalised patients. We do not know whether the post-COVID symptoms, including headache, may be related to other factors unique to hospitalisation, e.g., severity of illness, intubation, medication administration, or admission-related stress, and not just exposure to SARS-CoV-2 virus. Second, we included Caucasian, not African or Asian, participants; hence, ethnic differences could not be investigated. Third, we did not collect objective measures, e.g. blood oxygen saturation or other biomarkers, which could help to further characterise headache as a long-term post-COVID symptom. Fourth, data were collected telephonically. Headache diagnoses were based on the description provided by the patients, but this procedure has a bias well-known in survey studies. In particular, we did not have access to neuroimaging tests to look for secondary aetiologies other than COVID. Nevertheless, it should be noted that previous studies investigating post-COVID symptoms have used similar methods for data recruitment (11–14). Fifth, we collected data cross-sectionally and did not use a headache diary for assessing the evolution of headache. Therefore, we have not discriminated between patients who could have a NDPH-like headache and those who may have presented with a temporal pattern similar to that of tension-type headache. Likewise, we were not able to outline the evolution of headache throughout the follow-up period after hospital discharge, making it difficult to exclusively attribute the development of headache to COVID-19. We have also not collected data on treatments used and their effectiveness. It would be interesting to investigate when post-COVID headache appears and how it evolves to better characterise this potential “new” headache.

## Conclusions

This study reported that the presence of headache at hospitalisation was associated with tension-type like headache and persistent fatigue 7 months after hospitalisation. No differences in anxiety/depressive levels or sleep quality were observed between patients with and without headache at the acute phase. These results suggest the relevance of monitoring the headache as an onset symptom during the acute phase of COVID-19, due to its potential association with long-term post-COVID symptoms including post-COVID headache.

## Key Findings

1. The presence of headache in the acute phase of SARS-CoV-2 infection was associated with a higher prevalence of headache and fatigue as long-term post-COVID symptoms 7 months after hospital discharge.
2. Identification and recognition of headache as a symptom at the acute phase of COVID-19 could be used for monitoring the development of persistent headache as post-COVID sequelae.
